# Short and complex—Telomeres and genomes in CLL

**DOI:** 10.1111/bjh.70430

**Published:** 2026-03-11

**Authors:** Billy Michael Chelliah Jebaraj, Stephan Stilgenbauer

**Affiliations:** ^1^ Division of CLL, Department of Internal Medicine III University of Ulm Ulm Germany

**Keywords:** chronic lymphocytic leukaemia, complex karyotype, telomeres

## Abstract

Chronic lymphocytic leukaemia (CLL) with complex karyotype (CK) represents a high‐risk subgroup, often associated with refractory disease. In a patient cohort enriched for CK CLL, short telomeres were found to be associated with poor‐risk characteristics such as unmutated immunoglobulin heavy chain variable region (IGHV), del(11q)/del(17p), CK/genomic complexity (GC) and chromothripsis. The presence of short telomeres was associated with shorter time to first treatment (TTFT) in the full cohort and in cases with CK or del(17p), and in multivariable analysis as a prognostic factor, indicating the potential importance of this biomarker for CLL risk stratification.

Commentary on Ramos‐Campoy et al. Exploring the synergy between telomere length and genomic complexity in CLL. Br J Haematol 2026; 208:1121–1126.

Chronic lymphocytic leukaemia (CLL) exhibits a clinically heterogeneous disease course. While some patients experience indolent disease requiring no treatment for decades, others have aggressive disease progression, necessitating early intervention, with early treatment failure and reduced survival. Extensive efforts have been made to identify prognostic and predictive markers that stratify patients and guide therapeutic decisions. Accordingly, a number of clinical and genetic factors have been identified.^1^ CLL with complex karyotype (CK, ≥3 aberrations) constitutes a very high‐risk^2^ patient subgroup. Telomeres are nucleoprotein elements that protect the ends of chromosomes, thereby ensuring genomic stability. In CLL, different telomere components have been identified to be dysregulated. Critical shortening of telomeres can result in genomic instability and complex genomic rearrangements.^3^

In their paper, the authors study telomere length associations in CLL with CK in their work titled, ‘Exploring the synergy between telomere length and genomic complexity in CLL’.^4^ The authors analysed telomere length using quantitative real‐time polymerase chain reaction (qPCR) in 149 samples from treatment‐naïve patients. The cohort was enriched for CK (40.3%), and hence the median telomere length was shorter (2.82 kb) compared to representative CLL cohorts.^5^ Consistent with previous studies, the authors identified significant associations between short telomeres and high genomic complexity (≥3), presence of chromothripsis, presence of del(17p)/del(11q) and unmutated IGHV. Importantly, short telomere length (<median) was associated with shorter time to first treatment (TTFT) in the overall cohort and within cases with CK or del(17p). However, such a correlation was not observed within del(11q) or unmutated IGHV CLL subgroups.

CK has been described to be associated with resistance to chemotherapy as well as to ibrutinib‐ or venetoclax‐based treatments.^2^ However, the observation that telomere length provides additional prognostic discrimination within CK suggests that these patients represent a heterogeneous group. The finding that telomere length functions as an independent prognostic factor in multivariable analysis in this high‐risk cohort alongside IGHV mutation status and CK indicates that telomere length captures distinct biological information not fully encompassed by other biological disease features. Overall, telomere length may reflect cellular fitness and evolutionary capacity not adequately represented by individual genetic lesions or mutational patterns.

The association of short telomere length with CK and chromothripsis has been observed in CLL in the present and in earlier studies^4^, ^5^; however, it remains unclear whether telomere dysfunction is the cause of these poor‐risk aberrations or a consequence of greater proliferative history. The shorter TTFT of patients with CK suggests a more proliferative disease. Nevertheless, the biological role of telomere shortening in the acquisition of CK cannot be completely excluded in CLL. With the advent of methods to precisely evaluate lengths of individual telomere arms using single‐cell long‐read sequencing,^6^, ^7^ it is now possible to experimentally establish the temporal relationship between telomere dysfunction and genomic complexity in CLL. Analysis of serially collected CLL samples from timepoints before and after acquisition of CK could reveal whether critical shortening of telomere length of specific chromosomal arms precedes CK or chromothripsis of the same chromosomes. Conversely, deregulated DNA damage response or DNA repair pathways may also contribute to genomic complexity in CLL.^8^

Irrespective of its causal or consequential role in CLL pathogenesis, telomere length remains an underappreciated prognostic factor that may potentially even guide treatment decisions. The integration of telomere length into comprehensive risk stratification models warrants further investigation. It remains to be evaluated whether including telomere length in current prognostic scoring systems such as the CLL International Prognostic Index^9^ would improve the discriminatory power of these models. One of the key bottlenecks to employing telomere length for prognostic scoring in CLL is the variation in methods used across different laboratories for analysing telomere lengths. While the qPCR method used in the current study offers advantages as a high‐throughput system, the absolute telomere length value estimated by this method depends on the technique used for validation (single telomere length analysis [STELA] vs. terminal restriction fragment [TRF] analysis). Moreover, limited data are available in CLL on the comparability of telomere lengths analysed using different methods such as quantitative fluorescence in situ hybridization (Q‐FISH) or flow‐FISH or the more recent next‐generation sequencing‐based techniques.^10^ A universally adopted standardized and validated telomere length measurement method with stringent quality control procedures in a valid diagnostic laboratory setting may therefore be an important step towards employing telomere length for risk assessment and treatment decisions in CLL.

In conclusion, the present findings that telomere length is an independent prognostic factor even in this subgroup of high‐risk CLL reiterate the importance of considering telomere length for inclusion into clinical trials for CLL prognostication. Telomere length may eventually prove valuable for identifying patients requiring intensive monitoring and early intervention, and for informing decisions regarding the type and duration of treatment.

## AUTHOR CONTRIBUTIONS

B.M.C.J. and S.S. wrote the manuscript.

## CONFLICT OF INTEREST STATEMENT

B.M.C.J.: No competing financial interests; S.S.: personal fees and research support from AbbVie, Amgen, AstraZeneca, BeiGene, BMS, Galapagos, Gilead, GSK, Hoffmann‐La Roche, Johnson&Johnson, Lilly, Novartis, Sunesis, outside the submitted work.

## Data Availability

The data that support the findings of this study are available from the corresponding author upon reasonable request.
